# Israeli Acute Paralysis Virus: Epidemiology, Pathogenesis and Implications for Honey Bee Health

**DOI:** 10.1371/journal.ppat.1004261

**Published:** 2014-07-31

**Authors:** Yan Ping Chen, Jeffery S. Pettis, Miguel Corona, Wei Ping Chen, Cong Jun Li, Marla Spivak, P. Kirk Visscher, Gloria DeGrandi-Hoffman, Humberto Boncristiani, Yan Zhao, Dennis vanEngelsdorp, Keith Delaplane, Leellen Solter, Francis Drummond, Matthew Kramer, W. Ian Lipkin, Gustavo Palacios, Michele C. Hamilton, Barton Smith, Shao Kang Huang, Huo Qing Zheng, Ji Lian Li, Xuan Zhang, Ai Fen Zhou, Li You Wu, Ji Zhong Zhou, Myeong-L. Lee, Erica W. Teixeira, Zhi Guo Li, Jay D. Evans

**Affiliations:** 1 USDA-ARS Bee Research Laboratory, BARC-East Building, Beltsville, Maryland, United States of America; 2 Microarray Core Facility, National Institute of Diabetes and Digestive and Kidney Diseases, National Institutes of Health, Bethesda, Maryland, United States of America; 3 USDA-ARS Bovine Functional Genomic Laboratory, BARC-East Building, Beltsville, Maryland, United States of America; 4 Department of Entomology, University of Minnesota, St. Paul, Minnesota, United States of America; 5 Department of Entomology, University of California, Riverside, Riverside, California, United States of America; 6 USDA-ARS, Carl Hayden Bee Research Center, Tucson, Arizona, United States of America; 7 Department of Biology, University of North Carolina, Greensboro, Greensboro, North Carolina, United States of America; 8 USDA-ARS Molecular Plant Pathology Laboratory, Beltsville, Maryland, United States of America; 9 Department of Entomology, University of Maryland, College Park, Maryland, United States of America; 10 Department of Entomology, University of Georgia, Athens, Georgia, United States of America; 11 Illinois Natural History Survey, University of Illinois, Urbana, Illinois, United States of America; 12 School of Biology and Ecology, University of Maine, Orono, Maine, United States of America; 13 USDA-ARS Biometrical Consulting Services, Beltsville, Maryland, United States of America; 14 Center for Infection and Immunity, Mailman School of Public Health, Columbia University, New York, New York, United States of America; 15 National Center for Biodefense and Infectious Disease, George Mason University, Manassas, Virginia, United States of America; 16 College of Bee Science, Fujian Agriculture and Forestry University, Fuzhou, Fujian, People‚s Republic of China; 17 College of Animal Sciences, Zhejiang University, Hangzhou, Zhejiang, People‚s Republic of China; 18 Institute of Apicultural Research, Chinese Academy of Agricultural Science, Beijing, People‚s Republic of China; 19 Eastern Bee Research Institute, Yunnan Agricultural University, Kunming, People‚s Republic of China; 20 Institute for Environmental Genomics (IEG), University of Oklahoma, Norman, Oklahoma, United States of America; 21 Sericulture and Apiculture Department, National Academy of Agricultural Science, RDA Suwon, Republic of Korea; 22 Agência Paulista de Tecnologia dos Agronegócios/SAA-SP, Pindamonhangaba, São Paulo, Brazil; Stanford University, United States of America

## Abstract

*Israeli acute paralysis virus* (IAPV) is a widespread RNA virus of honey bees that has been linked with colony losses. Here we describe the transmission, prevalence, and genetic traits of this virus, along with host transcriptional responses to infections. Further, we present RNAi-based strategies for limiting an important mechanism used by IAPV to subvert host defenses. Our study shows that IAPV is established as a persistent infection in honey bee populations, likely enabled by both horizontal and vertical transmission pathways. The phenotypic differences in pathology among different strains of IAPV found globally may be due to high levels of standing genetic variation. Microarray profiles of host responses to IAPV infection revealed that mitochondrial function is the most significantly affected biological process, suggesting that viral infection causes significant disturbance in energy-related host processes. The expression of genes involved in immune pathways in adult bees indicates that IAPV infection triggers active immune responses. The evidence that silencing an IAPV-encoded putative suppressor of RNAi reduces IAPV replication suggests a functional assignment for a particular genomic region of IAPV and closely related viruses from the Family *Dicistroviridae*, and indicates a novel therapeutic strategy for limiting multiple honey bee viruses simultaneously and reducing colony losses due to viral diseases. We believe that the knowledge and insights gained from this study will provide a new platform for continuing studies of the IAPV–host interactions and have positive implications for disease management that will lead to mitigation of escalating honey bee colony losses worldwide.

## Author Summary

The mysterious outbreak of honey bee Colony Collapse Disorder (CCD) in the US in 2006–2007 has attracted massive media attention and created great concerns over the effects of various risk factors on bee health. Understanding the factors that are linked to the honey bee colony declines may provide insights for managing similar incidents in the future. We conducted this study to elucidate traits of a key honey bee virus, Israeli acute paralysis virus. We then developed an innovative strategy to control virus levels. The knowledge and insights gained from this study will have positive implications for bee disease management, helping to mitigate worldwide colony losses.

## Introduction

Honey bees are the most economically valuable pollinators of agricultural crops worldwide. In the U.S. alone, the value of agricultural crops pollinated by bees each year is more than $17 billion dollars [1]. In 2006, an enigmatic phenomenon labeled Colony Collapse Disorder (CCD) was observed in U.S. beekeeping operations. CCD is defined as an unusually sudden decrease in the numbers of worker honey bees, without expected signs of disease, starvation, or reproductive failure [2]. Such rapid declines have been observed throughout the history of beekeeping, and their causes often remain enigmatic. Since 2006, colony losses have been noted in beekeeping operations in much of the world [3], posing a significant threat to the pollination of many agricultural crops [4].

There is no single agent yet identified that causes CCD. Instead, it appears that CCD results from a combination of factors that include pathogens/parasites, pesticides, malnutrition, environmental stress, low genetic diversity, and migratory beekeeping practices. It is also conceivable that synergistic effects of two or more insults are behind recent declines. To that end, there is some evidence that interactions between pathogens and neuro-active pesticides can synergistically affect honey bee mortality, contributing to colony depopulation [5], [6].

An early survey [7] of healthy and CCD-affected colonies in the U.S. found a significant correlation between CCD-affected colonies and *Israeli acute paralysis virus* (IAPV), an RNA virus first identified in 2004 [8]. The result drew immediately international attention to the risks of virus infection in honey bees. The role of IAPV in triggering colony declines, alone or in concert with other factors, remains a research priority. The parasitic mite *Varroa destructor* has long been considered the primary threat to honey bees [9], in part because this mites serves as a vector of honey bee viruses [10]. For example, levels of *Deformed wing virus* (DWV), a common virus that has killed billions of honey bees across the globe, are greatly increased following *Varroa* transmission [11]. A recent study showed that *Varroa* mites can also serve as vectors of IAPV; furthermore, the mite/virus association was shown to reduce host immunity and promote elevated levels of IAPV replication [12], providing more evidence for the damaging effects of viruses associated with *Varroa* mite infestations.

In this study, we investigated the molecular basis of pathogenesis, transmission and genetic diversity of IAPV in honey bees and evaluated the impacts of IAPV infection on colony losses. We also determined the global transcriptional profiles of honey bee responses to viral infection. Finally, we examined the inhibitory effect of small interfering RNA (siRNA) that targets putative virus-encoded proteins (VSR) on IAPV replication. The replication of single-stranded positive-sense RNA viruses results in the synthesis of complementary negative-stranded RNA, thereby producing dsRNA replicative intermediates that are attractive targets for defenses based on RNA interference. To counteract host RNAi antiviral defense, viruses have evolved strategies to suppress the antiviral effects of RNAi. A recent study with *Cricket paralysis virus* (CrPV) showed that the sequences upstream of a highly conserved sequence (DVEXNPGP) within the N-terminal region of CrPV ORF-1 encode a potent suppressor that mutes the RNAi antiviral defense in *Drosophila* [13]. As a result, we speculated that IAPV may possess a similar mechanism to counteract the antiviral response of hosts. We believe that knowledge gained from this study will lead to better understanding of the dynamics of virus disease pathogenesis in honey bees and help mitigate escalating colony losses worldwide.

## Results

### IAPV attacks every stage and caste of honey bees and causes systemic infection in honey bees

**Figure 1:**
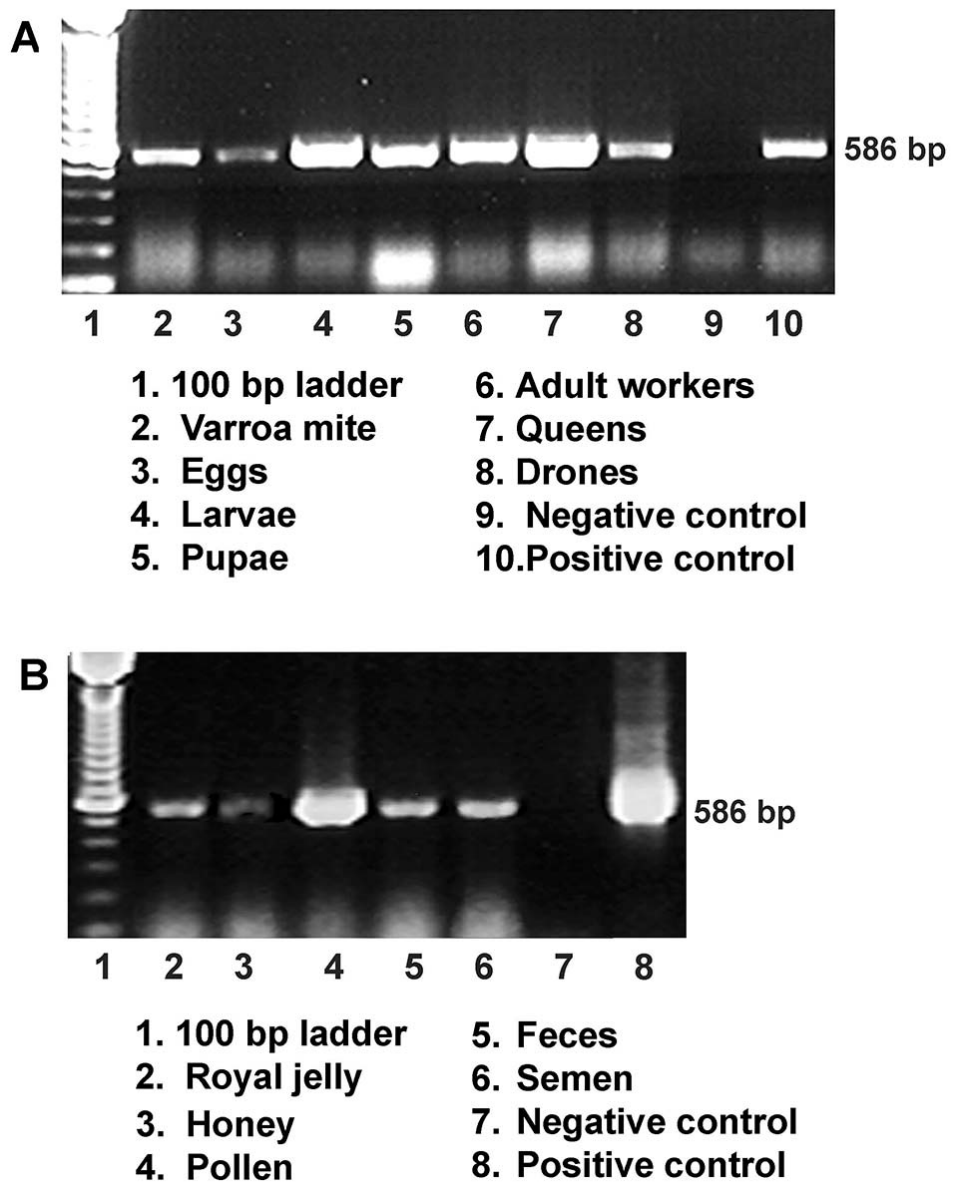
Detection of IAPV infection in a representative honey bee colony. (**A**) Gel electrophoresis of RT-PCR amplification for specific detection of IAPV from samples of worker eggs, worker larvae, worker pupae, adult workers, drones, queens and parasitic mites, *Varroa destructor* collected from the same colony. (**B**) Gel electrophoresis of RT-PCR amplification for specific detection of IAPV from samples of colony foods, queen feces, and drone semen. For both A and B, a PCR band of 586 bp indicating the IAPV infection is observed in examined samples.

**Figure 2:**
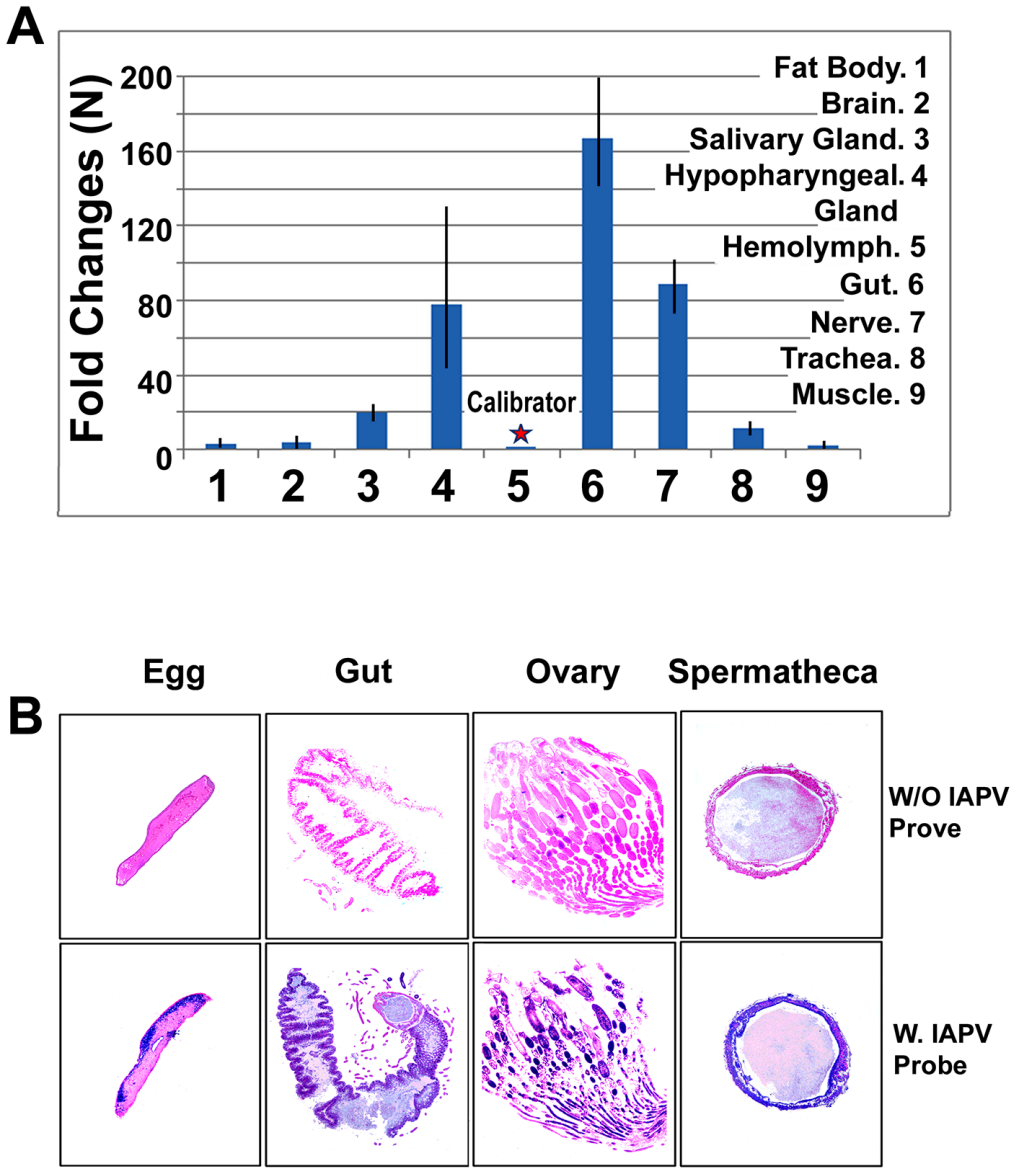
Relative abundance of negative strand RNA of IAPV genome copies in different tissues of honey bees and in situ hybridization analysis of queen somatic and germ tissues. (**A**) The hemolymph harbored the minimal level of IAPV and therefore was chosen as a calibrator. The concentration of negative strand RNA of IAPV in other tissues was compared with the calibrator and expressed as n-fold change. The y-axis depicts fold change relative to the calibrator. (**B**) The slides were not hybridized with DIG-labeled IAPV probe (top row, negative control) and the slides were hybridized with DIG-labeled IAPV probe (bottom row). Positive signal is dark blue to purple and the negative areas are pink in color. The infected tissues of queen gut, ovary, spermatheca and queen eggs are indicated by a dark blue/purple color.

Although the bee colonies in this study showed no clinical signs of infection, IAPV was found widely in surveyed honey bees colonies. IAPV-positive PCR signal was detected in eggs, larvae, pupae, adult workers, drones, and queens as well as *V. destructor* that fed on the bees (Figure 1A). In addition, IAPV-specific PCR signal was also detected in royal jelly, honey, pollen, queen feces and drone semen collected from IAPV positive colonies (Figure 1B). Strand specific RT-qPCR assays revealed that IAPV causes systemic infection in honey bees. IAPV replication was detected in hemolymph, brain, fat body, salivary gland, hypopharyngeal gland, gut, nerve, trachea, and muscle. However, the relative abundance of negative stranded RNA copies of IAPV in the different tissues varied significantly. The hemolymph (i.e., hemocytes) harbored the lowest level of IAPV among the examined tissues and therefore was chosen as the calibrator. The difference in IAPV abundance in other tissues relative to hemolymph ranged from 2.23- to 167-fold in the following order from lowest to highest concentration: muscle<fat body<brain<trachea<salivary gland<hypopharyngeal gland<nerve<gut (Figure 2A). *In situ* hybridization showed IAPV specific signals localized in egg, gut, ovaries, and spermatheca of infected queens.

### Colony traits and IAPV infection

**Figure 3:**
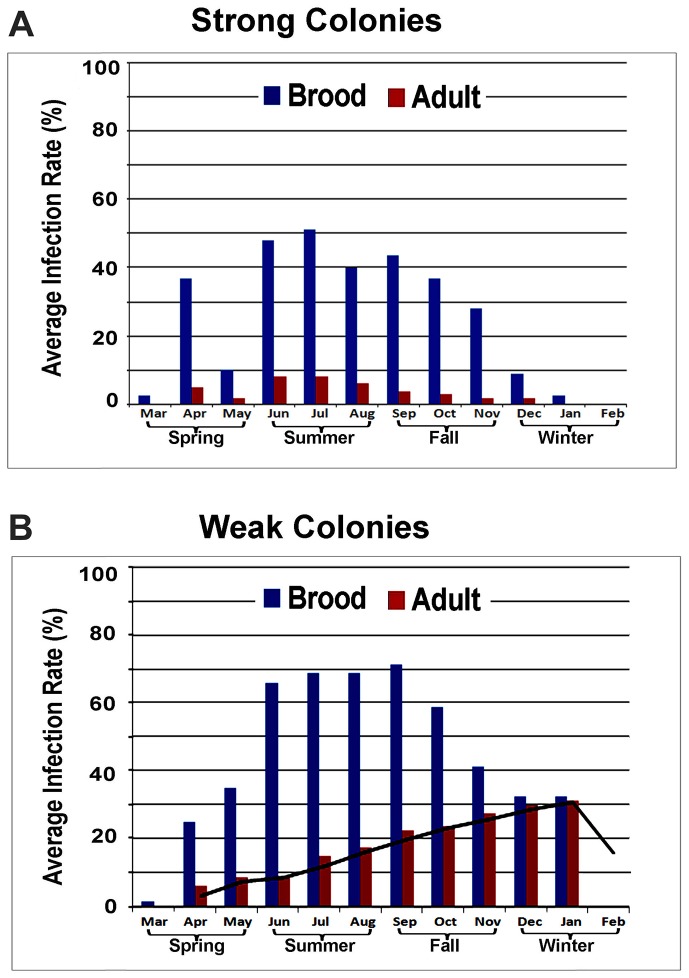
Average prevalence of IAPV infection in a single month. (**A**) Strong colonies. (**B**) Weak colonies. For both strong and weak colonies, the prevalence of IAPV infection in the brood was significantly higher than in adult bees. While strong colonies did not exhibit significant seasonal variation in IAPV infection, the infection rate of IAPV in adult bees in weak colonies increased from Spring to Summer and Fall and peaked in the Winter. All strong colonies survived through the cold winter months while the weak colonies collapsed before February.

IAPV was found to be the third most common virus infection in bee colonies after DWV and Black Queen Cell Virus (BQCV). Over the 4-year study period, the infection IAPV detected in the brood was significantly higher than in adult bees (p<0.001). When we divided our experimental bee colonies into those with more than ten frames covered with adult workers and more than six frames filled with brood and food stores (‘strong’) versus those with fewer than ten frames of adult bees, less than six combs with brood and small patches of food stores (‘weak’), we found a measurable difference in IAPV infection levels. The average rate of IAPV infection per month was 49% for brood and 19.5% for adults in weak colonies and 26% for brood and 3.25% for adults in strong colonies. The overall rate of IAPV infection in weak colonies was significantly higher than in the strong colonies (p<0.01 for brood and p<0.001 for adults). While no statistically significant seasonal variation in IAPV infection was observed in the strong colonies, the infection rate of IAPV in adult bees in weak colonies increased from spring to summer and fall and peaked in winter. While strong colonies in our survey survived through the cold winter months, almost all weak colonies collapsed before February (Figure 3). While strong colonies in our survey survived through the cold winter months, almost all weak colonies collapsed before February (Figure 3).

### High genetic diversity exists between different strains of IAPV

**Figure 4:**
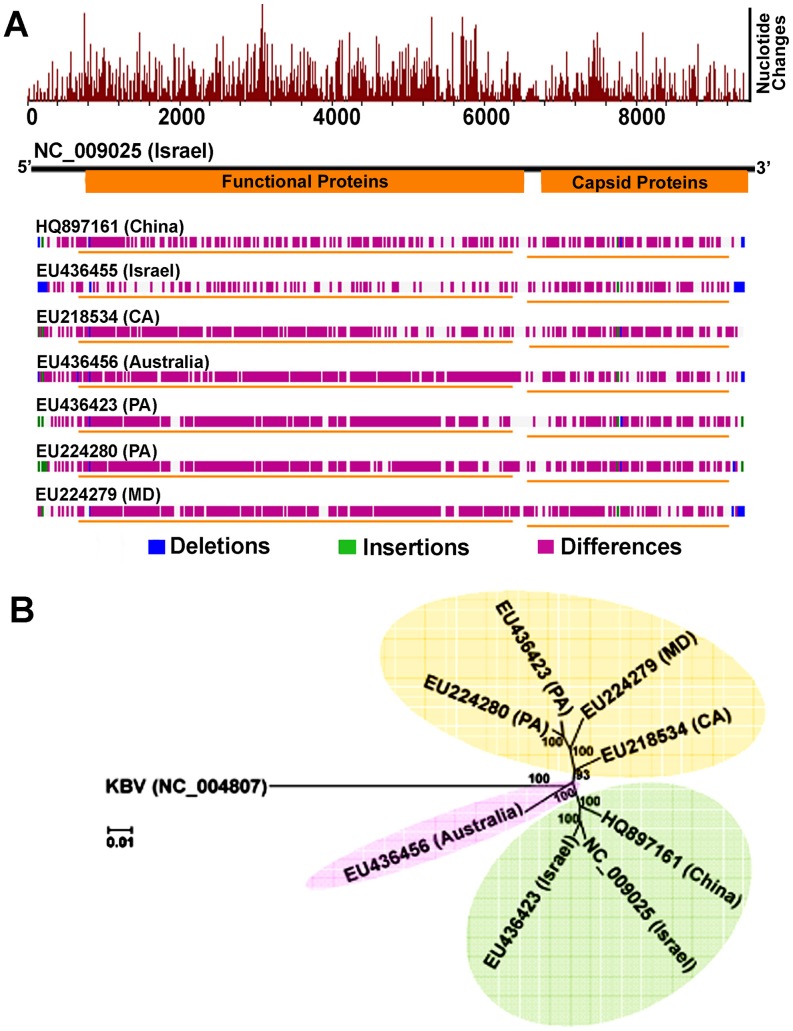
Genome-wide sequence diversity and phylogenetic relationship of IAPV isolates. (**A**) A graphical representation of the pair-wise global alignments of the reference sequence of IAPV (NC_009025), the first complete sequence of IAPV, with other IAPV genome sequences individually. This figure is retrieved from GenBank and modified. The alignments were pre-computed using the “band” version of the Needleman-Wunsch algorithm. The top histogram shows the average density of nucleotide changes (excluding gaps, insertions and undetermined nucleotides) in all additional sequences per a reference sequence segment. The length of the segment is equal to the length of the reference sequence divided by the width of its graphical representation (in pixels). The deletions, insertions and differences among the sequences are highlighted in blue, green and red-violet, respectively. If no significant alignment could be obtained for a particular sequence, no horizontal bar is shown. (**B**) Phylogenetic tree showing the relationship of IAPV strains from different geographic locations globally. Numbers at each node represent bootstrap values as percentages of 500. Individual sequences are labeled with their GenBank accession numbers.

The complete genomes of IAPV strains collected in the US states of Maryland, California, and Pennsylvania were obtained by direct sequencing of overlapping RT-PCR fragments and partial sequences from both 5′UTR and 3′UTR and deposited in GenBank with accession numbers, EU224279, EU218534, and EU224280, respectively. Comparison of US, Chinese and Australian IAPV strains with the first reported Israeli IAPV strain at the genome level showed a significant genetic divergence among different strains, providing evidence of quasi-species dynamics in IAPV populations. The polymorphisms in IAPV were found more frequently in 5′ UTR and functional protein coding regions compared to the capsid protein coding region and 3′ UTR (Figure 4A). Phylogenetic analysis using full-length viral genomes showed that the Australian IAPV strain constitutes the earliest lineage of the phylogenetic tree. The US strains branch to form a distinct lineage distantly related to the Israeli and Chinese strains of IAPV (Figure 4B).

### IAPV infection results in more significant changes in gene expression in adult bees than in brood

The results of microarray analyses yielded a large group of differentially expressed genes. The principal component analysis (PCA) mapping showed that the total accumulative variance of the first three PCs was 78% for adult and 67.4% for brood, respectively, and suggested that two kinds of experimental populations (IAPV positive vs IAPV negative) were well separated for both adults and brood. The cluster analysis showed overall similar data patterns (Figure S3A and B), indicating that inter-individual differences had a minimum effect on gene expression data. The treatment variance (IAPV-infected versus uninfected) was significantly higher than error variance for both adult bees and brood (both p<0.01) (Figure S3C). This confirmed that variation among samples was largely due to IAPV and suggested the good data quality for two ANOVA data analysis in both adults and brood. The distribution of differentially expressed genes in both adults and brood are presented by volcano plots (Figure S3D). All microarray data were deposited in the NCBI public database with accession number GSE46278.

**Figure 5:**
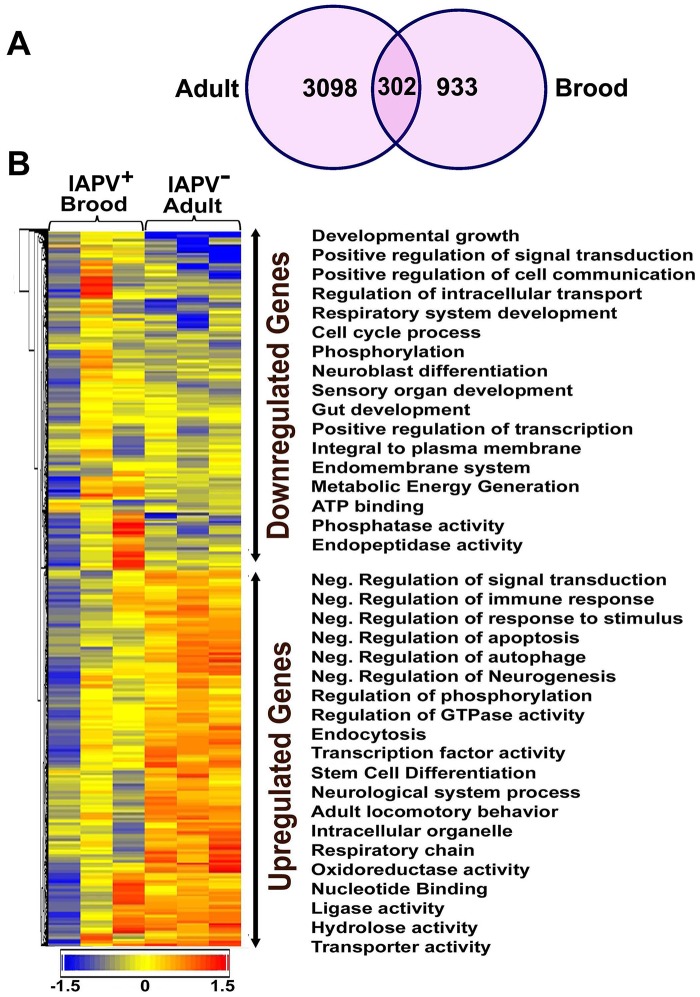
An overview of gene expression profiles in IAPV infected adults and brood. (**A**) Venn graph compares regulated genes between adult and brood. The intersecting circles indicate overlapping genes between adult and brood. Of 4615 genes with altered expression in IAPV-positive adult and 1350 genes with altered expression in IAPV-positive brood, the number of overlapping genes between adults and brood was 336. (**B**) A heat map illustrates differential expression profiles of up- and down- regulated genes for adults and brood. The number of genes with altered expression was significantly higher in IAPV infected adult than in IAPV infected brood. The relative levels of gene expression are depicted using a color scale where blue indicates the lowest and red indicates the highest level of expression. Significantly enriched Gene Ontology (GO) terms of up- and down regulated gene clusters inducted by IAPV infection (ρ≤0.05) appear on the right side of the heat map.

Overall, the transcriptional response to IAPV infection was substantially different between adults and brood. There were 2,522 up-regulated and 2,093 down-regulated genes identified in IAPV-positive adults, but only 825 up-regulated and 525 down-regulated genes identified in IAPV-positive brood with a very small fraction of overlapping genes between the two groups (Figure 5A). Of the up-regulated and down-regulated genes, overlapping genes between adult and brood were 268 and 68, respectively. A heat map illustrates the differential expression of enriched functional genes between adults and brood (Figure 5B). Of the genes transcriptionally altered by IAPV infection, 2,150 genes identified in adults and 716 genes identified in brood could be assigned a putative function based on orthology to *D. melanogaster* genes. The GO-enriched analysis of the genes that displayed fold-changes of more than 1.5 (False Discovery Rate adjusted ρ value≤0.05) and had putative *D. melanogaster* orthologs by the Database for Annotation, Visualization and Integrated Discovery (DAVID) revealed major functional clusters including metabolism, host cell transcription, signal transduction, cell cycle, hormone synthesis, endocytosis, phagocytosis, autophagy, and innate immune response (Table S1 and S2). The majority of genes with up-regulated expression were related to the regulation of signaling transduction and immune response, while the majority of those with down-regulated expression were involved with metabolic energy generation. Of the top functional clusters, genes that were related to immune response functions were of particular interest in this study.

**Figure 6:**
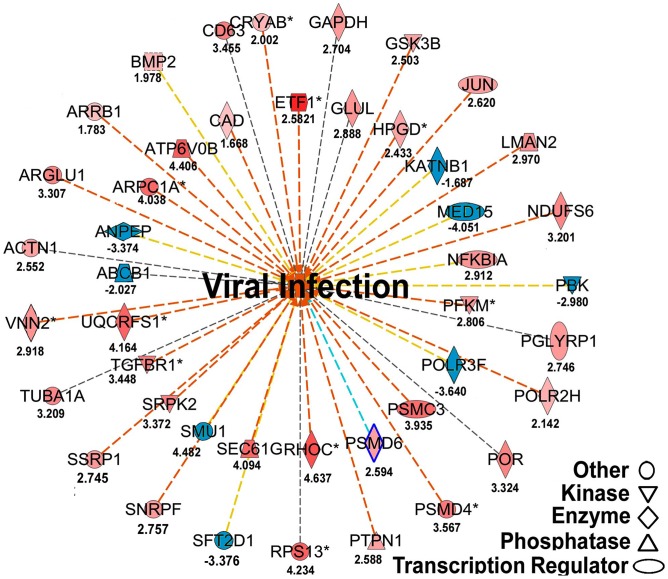
Regulated molecules that are involved in host metabolism and immunity. The figure illustrates a network predicted by Ingenuity Pathway Analysis that is centered by viral infection and associated with molecules involved in host energy metabolism and immunity. Solid and dashed connecting lines indicate the presence of direct and indirect interactions, respectively. Nodes indicate input of genes into the pathway analysis and the different symbols indicate gene functions (Legend in bottom left). The intensity of the node color-(red) indicates the degree of up-regulation while the intensity of the node color-(green) indicates the degree of down-regulation. The numbers shown in each node indicates the fold change in response to IAPV infection.

We examined the integrated networks and pathways of genes that were up- and down- regulated in response to IAPV infection. The global canonical pathway analysis of 2,150 genes identified in adults using the Ingenuity Pathway Knowledge Base led to identification of five top canonical pathways, including mitochondrial dysfunction, TCA cycle II, protein ubiquitination pathway, eIF2 signaling and γ-glutamyl cycle, with mitochondrial dysfunction (37 molecules, ρ-value 3.93E-17) as the most significantly affected pathway. Among five significantly disturbed canonical pathways, four showed significant up-regulation and only the γ-glutamyl cycle pathway showed significant down-regulation. The analysis of 716 IAPV regulated genes in brood identified five top canonical pathways, eIF2 signaling, mitochondrial dysfunction, mTOR signaling, TCA cycle II, regulation of eIF4 and P70S6K signaling, with eIF2 signaling (25 molecules, ρ-value 6.15E-16) as the most significantly affected canonical pathway. All pathways showed significant up-regulation. Among 25 networks identified, one was centered by viral infection in adults (Figure 6) and contains both up- and down- regulated genes that are involved in pathways related to host defense responses such as oxidative phosphorylation, ABC transporter, endocytosis, phagocytosis, TGF-beta signaling pathway, mTOR signaling pathway, MAPK signaling pathway, JAK-STAT pathway, and lysosome.

**Figure 7:**
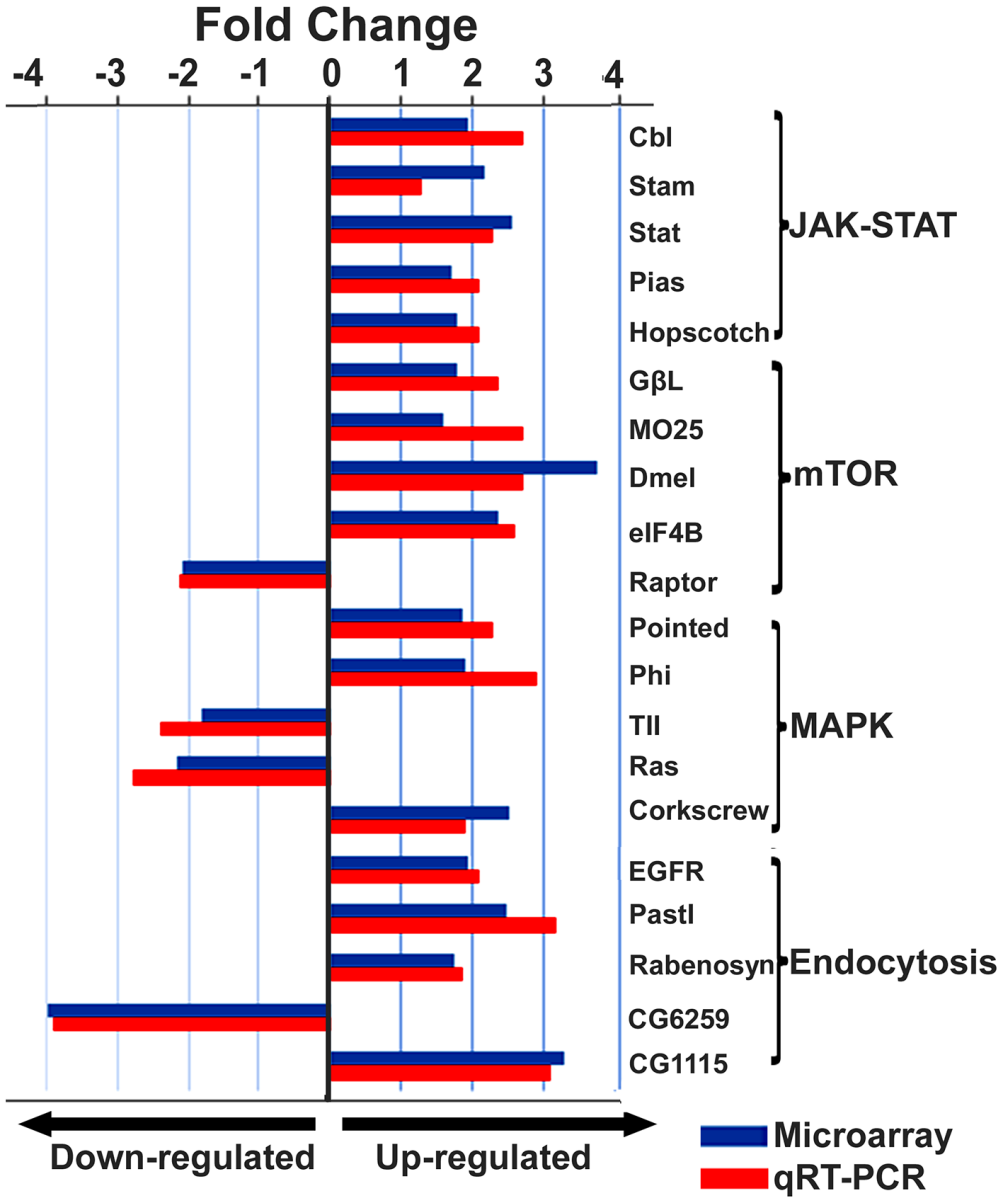
Expression levels of immune-related transcripts in IAPV infected adults. The expression levels of genes that were assigned to JAK-STAT, mTOR, MAPK and Endocytosis pathways were measured by microarray analysis and further confirmed using TaqMan RT-qPCR. The expression results obtained from microarray and qRT-PCR analyses showed good alignment.

IAPV infection triggers multiple immune signaling in adult bees. qRT-PCR confirmation of immune related genes showed the components of the Janus Kinase/Signal Transducers and Activators of Transcription (JAK-STAT) pathway including Cbl, STAT, PIAS, and Hopscotch had ≤2 fold elevated expression in response to IAPV infection. The components of Mammalian Target of Rapamycin (mTOR) signaling pathway including GβL, MO25, Dmel, and eIF4B had ≤2 fold elevated expression in response to IAPV infection. The expression of genes including Pointed, Phi, and Corkscrew that had functional association with Mitogen-activated Protein Kinases (MAPK) pathway was upregulated to 2.3-, 2.91- and 1.92-fold respectively, in response to IAPV infection. The expression of genes EGFR, PastI, Rabenosysn, and CG1115, involved in endocytosis was also upregulated by 2.1-, 3.18-, 1.88-, and 3.1- fold, respectively. IAPV infection also caused the down-regulation of mTOR pathway gene such as Raptor, MAPK pathway genes, TII and Ras, and endocytosis gene CG6259 ranging from −2.14 to −3.9 fold. qRT-PCR analysis of immune related genes in IAPV-infected adults showed considerable concordance with the normalized microarray data (Figure 7).

### Identification of a putative viral interference protein

**Figure 8:**
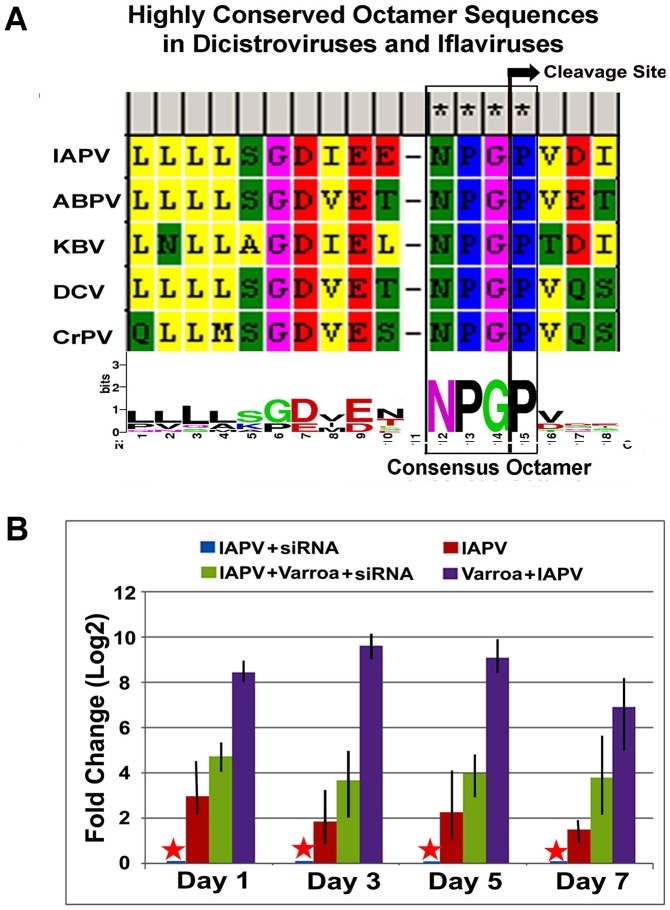
IAPV-encoded putative suppressor of RNAi. (**A**) Highly conserved octamer sequences identified in dicistroviruses. A putative viral suppressor of RNAi (VSR) is presumably located upstream of DvExNPGP. The cleavage site between the glycine and proline is marked by an arrow. (**B**) Quantitative analysis of the effects of silencing putative VSR on IAPV replication. The amount of negative stranded RNA of IAPV was measured by RT-qPCR, normalized to the corresponding β-actin in the same sample. The data shown represent the mean value for three separate experiments. Error bars represent the range of fold change.

The sequence motif of D_I_E_E_NPGP was identified in the N-terminal region of ORF-1of IAPV and other members of the *Dicistroviridae* family infecting honey bees such as KBV, and ABPV, where the uppercase letters of the sequence motif indicates residues with absolute sequence conservation (Figure 8A). An RNAi-mediated knockdown experiment showed that silencing putative VSR in IAPV genome could effectively inhibit replication of IAPV and confer significant antiviral activity in honey bees. Quantification of the titer of negative strand RNA of IAPV showed that feeding siRNA resulted in a remarkable reduction in IAPV replication. The bees in Group I (*IAPV+siRNA*) had the lowest IAPV titer among four experimental groups at all time points (days 1, 3, 5 and 7) and this group was therefore chosen as a calibrator. Compared to the calibrator, Group-II (*IAPV*), Group III (*IAPV+Varroa+siRNA*), and Group IV (*IAPV+Varroa*) averaged 4.78±0.25, 17.5±0.56, and 451.5±2.72 (Mean±SE, N = 3) folder higher titers of negative strand RNA of IAPV, respectively. The significant reduction in virus replication observed in Groups-I and III at day 1 post treatment indicated that the impact of siRNA on the virus life cycle takes place within 24 hours. There was no significant difference in virus titer among different time points for each group (ρ<0.05, ANOVA). The highest titer of virus replication seen in Group IV challenged by *V. destructor* with no siRNA treatment provides additional evidence for the role of *V. destructor* in virus transmission and activation in honey bees (Figure 8B). The antiviral effects of siRNA from this study (siRNA-_suppressor_) were compared to those of siRNA targeting the 5′ Internal Ribosomal Entry Site (IRES) of IAPV (siRNA-_5′IRES_) that was shown to confer antiviral activity in bees in our previous study [14]. The virus titer in bees fed siRNA-_5′IRES_ was 3.3±0.54, 4.5±0.33, 3.9±0.21 and 5.2±0.67 (Mean±SE, N = 3) fold higher than the group fed with siRNA-_suppressor_ at Day 3, Day 5, and Day 7 post treatment, respectively. However, no significant difference (p valve>0.05) was observed between groups received dsiRNAs targeting different genomic regions when Varroa mites were introduced.

## Discussion

The association of IAPV with honey bee declines has led to an increased awareness of the risks of viral infections on bee health. In this paper, we present a long-term study of the biological and molecular features of IAPV infection in honey bees. Our results showed that IAPV is established as a persistent infection in honey bee population and infects all developmental stages and different sexes of honey bees. The tissue tropism study showed that IAPv replicates within all bee tissues but tends to concentrate in gut and nerve tissues and in the hypopharyngeal glands. The highest titer of IAPV was observed in gut tissues and, in conjunction with detection of IAPV in colony food, suggests that food serves as a vehicle for within-colony horizontal transmission. The next highest titer of IAPV replication was observed in nerve tissue and indicates tropism of IAPV to the bee nervous system, consistent with observed pathologies. Specifically, while IAPV-infected bees in our study remained asymptomatic, infected bees can exhibit shivering wings and progressive paralysis, typical symptoms of nerve-function impairment [8]. The third highest titer of IAPV was identified in hypopharyngeal glands and may explain the presence of the virus in royal jelly, a product synthesized in these glands and fed to queens and larvae. Royal jelly, along with nectars shared among adult workers, thus provide an important route for viral movement within the colony.

A previous study showed that honey bees became infected with IAPV after exposure to *V. destructor* that carried the virus [12], illustrating vector-mediated horizontal transmission. In addition, the detection of IAPV in the digestive tracts and feces of queen bees along with detection of the virus in colony food supplies suggest a food-borne transmission pathway, arguably driven by frequent trophallaxis (mouth-to-mouth sharing of food) between colony members. The detection of IAPV in eggs and larvae not exposed to *V. destructor* that serves as a vector to facilitate the horizontal transmission of the virus to their honey bee hosts, together with detection of IAPV in queen ovaries suggests a vertical transmission pathway from queens to their progeny. Further, the detection of IAPV in drone semen, and in the spermatheca used to store sperm in queens for fertilizing eggs, suggests that venereal (sexual) infection is another plausible mechanism by which this virus is transmitted. We suspect that IAPV manifests itself in a way similar to the iflavirus Deformed wing virus. Namely, when colonies are healthy, the virus persists via vertical transmission and exists in a latent state without perturbing host immunity. When honey bees live under stressful conditions such as *Varroa* mite infestation and overwintering stress, the virus replicates quickly and becomes more infectious, leading to the death of hosts and possible collapse of the colony.

RNA viruses are characterized by their quasi-species population structure, that is, clouds of genetically related variants that collectively determine pathological characteristics of the population [15]. Genome analyses of IAPV strains shows several lineages. Previous genetic analysis of IAPV suggested the existence of at least three distinct IAPV lineages, two of them present in the US [16]. Our phylogenetic analysis confirmed this finding but showed a long period of independent evolution of IAPV strains in different collections. Genetic variation may account for the difference in virulence properties and severity of disease manifestations among IAPV strains and in fact, Cornman et al. [17] noted an especially high rate of nucleotide divergence among IAPV isolates sequenced from heavily impacted populations. Future studies using a combination of genome sequencing and single-nucleotide polymorphism analyses based on sequencing RNA pools (deMiranda et al. 2010, Cornman et al., 2013), should provide more insights into the evolutionary history, functional variation, and pathogenicity of this virus.

The rate of IAPV infection in brood was higher than in adult bees for both strong and weak colonies. IAPV infection triggers a more profound alteration of gene expression in adult bees than in brood, shown by the fact that the number of genes with altered expression was four times higher in adults than in brood. The gene expression data did not provide obvious clues to the molecular mechanism(s) underlying the maintenance of the viral latency in brood. Genes involved in immune response showed no clear trend in expression in IAPV-positive brood. Genes involved in host immunity were significantly invoked in IAPV-infected adults, indicating that IAPV infection triggers active immune responses in adult bees. The transition of the virus from latency to activation of host immune response was likely triggered by exogenous stressors that affect bees at the adult stage. The evidence that mitochondrial dysfunction was the most significantly affected canonical pathway in IAPA-infected adults suggests that IAPV likely caused pathogenesis of energy-related host processes and functions, a condition that tends to worsen host nutritional status and impair host defenses mechanisms [18]. JAK-STAT was reported to be involved in the control of the viral load in DCV-infected *Drosophila* [19]. Components of the JAK-STAT pathway were up-regulated in response to IAPV infection. Other signaling cascades such as mTOR and MAPK pathways reported to be involved in antiviral immune responses [20], [21], also showed expression changes in response to the IAPV infection. However, components of the Toll and Imd signaling pathways, implicated in antiviral immunity in insects [22], [23] were not up-regulated by IAPV infection. Toll and Imd are not always linked with antiviral immunity and, in particular, these pathways were not a factor during infection of *D. melanogaster* by *Drosophila* C virus, a relatively close relative of IAPV [19], suggesting that different viruses trigger distinct antiviral responses. Knowing which pathways respond specifically to viral infections will enable more targeted pharmacological or genetic control strategies.

Our results show that silencing a putative immune-suppressive protein encoded by IAPV led to significant reduction in IAPV replication without detrimental effect on bee hosts. This suggests that IAPV may also encode an RNAi suppressor. RNAi technology has been employed in previous work to combat virus infection in honey bees. The injection and feeding of Remebee, a dsRNA homologous to IAPV has proven effective in not only reducing the intensity of IAPV infection in honey bees [24], but also strengthening honey bee colonies [25]. A recent study showed that the feeding of siRNA targeting an Internal Ribosomal Entry Site (IRES) of IAPV required for protein translation could confer antiviral activity in bees [14]. That feeding siRNAs targeting VSR in this study led to suppressed IAPV replication reinforces the therapeutic potential of RNAi for treatment of viral diseases in honey bees, by showing that carefully designed constructs can temper a potent counter-response to the host immune system. Further exploration of antiviral effects of putative suppressors of RNAi of other bee viruses such as KBV and ABPV, which share the same sequence motif of D_I_E_E_NPGP with IAPV, is warranted.

IAPV has a longstanding presence in managed honey bees [26]. While IAPV is not consistently tied to CCD, its ability to cause increased mortality in honey bees has been firmly established. Our results showed that host health status and environmental conditions indeed play a critical role in IAPV infection dynamics. While the simultaneous presence of multiple viruses in honey bees makes Koch's postulates of disease causality difficult to fulfill [27], the presence and diversity of viruses in bee colonies has high predictive value for colony mortality [28]. The negative correlation between the level of IAPV infections and the size of host populations, in combination with other stress factors, has significant negative impact on colony survival and is likely a contributing factor to poor winter survivorship of honey bee colonies. The present study provides an improved starting point for continuing studies of the virus-host interactions and for efforts to formulate strategies to reduce colony losses due to viral diseases.

## Materials and Methods

### Bee samples

A brood frame containing bee samples of various ages and food stores was removed from each of three declining colonies colony selected from each colony maintained in a northern California queen-breeding operation in Spring. Honey bees (*Apis mellifera ligustica*) of different developmental stages and sexes (eggs, larvae, pupae, workers, adult drones, and queens) and colony foods (honey, pollen, and royal jelly), as well as parasitic mites, *V. destructor*, were sampled for the detection of IAPV infection using RT-PCR method. Clear fecal material, 20–25 µl per queen, was collected by isolating queens individually in a 100×15 mm petri dish for approximately 30 minutes to allow them to defecate. Approximately 20–25 ul of semen was also collected from 25 drones of each colony.

### Tissue dissection

To determine the ability of IAVP to spread and replicate within honey bee hosts, fifteen adult worker bees were collected from each of the three colonies maintained placed in two USDA Bee Research Laboratory apiaries in Beltsville, MD and identified to be IAPV positive and subjected to tissue dissection. Under a dissecting microscope, each worker was fixed on the wax top of a dissecting dish with steel insect pins and 10–15 µl of hemolymph was micropipetted from a small hole made with a sterile needle on the dorsal thorax. Following hemolymph collection, a dorsal mid-line cut was made from the tip of the abdomen to the head with scissors, and tissues including hemolymph, fat body, brain, salivary gland, hypopharyngeal gland, gut, nerve, trachea, and muscle were individually removed from each worker. The scissors and forceps were cleaned between dissections with a cotton pad soaked with 10% bleach (0.003 sodium hypochlorite) and another soaked with 70% alcohol, followed by a final rinse in sterile water. To prevent contamination with hemolymph, all tissues were rinsed once in 1× phosphate-buffered saline (PBS) and twice in nuclease-free water. The washing solution was changed after each tissue collection to prevent cross-contamination. The same tissues of different bees of the same colony were pooled together for subsequent RNA extraction. All freshly dissected tissues were immediately subjected to RNA extraction and then stored in −80°C freezer in the presence Invitrogen RNaseOUT Recombinant Ribonuclease Inhibitor until quantitative examination of the tissue tropism by strand-specific TaqMan quantitative RT-PCR (RT-qPCR).

Additionally, twenty eggs were also collected from the colonies identified to be IAPV positive using a fine brush. The eggs were washed in 5% bleach solution for five minutes then rinsed in sterile water to eliminate surface contamination of the virus [29]. Queens from the same IAPV-positive colonies were collected and tissues of gut, ovaries and spermatheca were excised following the methods described above. Both eggs and queen tissues were fixed in 4% paraformaldehyde in 100 mM PBS (pH 7.0), then stored in 70% ethanol (200 Proof) at 4°C until in situ hybridization (ISH) assays for localization of the virus.

### Virus seasonality

To determine seasonal activities and impacts of IAPV on honey bee health, samples of adult workers and brood (4^th^ and 5^th^ instar mature larvae, prepupae, and white-eyed pupae) of *A. mellifera ligustica* were collected from ten bee colonies maintained in apiaries of the USDA Beltsville Bee Research Laboratory from March 2008 to February 2012 and were subject to RT-PCR assay for presence of IAPV. The experimental colonies were divided into healthy and weak colonies based on the size of adult populations, amount of sealed brood, and presence of food stores. 20 adult workers and 20 unsealed brood were collected individually from each of five strong and five weak colonies every month and examined for the virus infection individually.

For each colony, the rate of the virus infection and strength of individual colonies were recorded every month and the infection rate was calculated based on percentage of tested bees (adult or brood) that were infected (N = 20). The average infection rate each month for both strong and weak colonies was calculated by combining the date from five colonies each month and four years of the same month (N = 5×4). The infection rates of IAPV were compared for colony strength (healthy vs. weak), developmental stages (adult vs. brood) and months of the year. Because the data are binomial in nature (for each sample, the number of uninfected of 40 total), analysis was based on a generalized linear mixed model (because random effects were included), using the logit link and the R software (R Core Team 2012) with the lme4 package [30]. The combination of lowest AIC and main effects retention (i.e. preserve main effects in the model even if not significant as long as higher order terms involving these main effects were significant) was used to select a model that captured the important features of the data.

### Total RNA extraction

Invitrogen Trizol reagent was used for isolation of total RNA from whole bees and bee tissues, as well as from colony foods, queen feces, drone semen, and *Varroa* mites, in accordance with the manufacturer's instructions. After confirmation of IAPA positive status by RT-PCR, total RNAs intended for microarray analysis were further purified with Qiagen RNeasy Microarray Tissue Mini Kit. RNA integrity was assessed with a 2100 Bioanalyzer system (Agilent Technologies, Palo Alto, CA) and RNA Lab Chip. Only samples with an RNA integrity number (RIN) of 6 or more were used [31].

### RT-PCR and strand specific RT-qPCR

The Promega one-step access RT-PCR system (Madison, WI) was used for IAPV detection as previously described [32]. Negative and positive controls (previously identified positive sample) were included in each run of RT-PCR reaction. The specificity of the amplified products was confirmed by sequence analysis of PCR products.

RNA samples extracted from different tissues of adult workers were analyzed for the abundance of negative-stranded RNA, a replicative intermediate form of positive strand RNA viruses, using strand-specific reverse transcription coupled with TaqMan quantitative PCR (RT-qPCR) [33], [34]. For each tissue sample, the first strand of cDNA was synthesized from total RNA using Superscript III reverse transcriptase (Invitrogen) with Tag-sense primer, Tag-IAPV-F1 (5′-AGCCTGCGCACGTGG gcggagaatataaggctcag -3), where the capitalized sequences of Tag were published by Yue and Genersch [35]. The resulting synthesized cDNAs were then purified using MinElute PCR purification kit (Qiagen) followed by MinElute Reaction Clean kit (Qiagen) to remove short fragments of oligonucleotides and residue of enzymatic reagents to prevent amplification of non-strand specific products [34]. The resulting cDNA derived from negative stranded RNA was amplified using the Platinum Taq High Fidelity Polymerase (Invitrogen) in a 25-ul reaction containing 2 µl cDNA, 0.25 µM of Tag primer (3′-AGCCTGCGCACCGTGG- 5′), 0.25 µM of antisense primer, IAPV-R1 (5′-cttgcaagataagaaaggggg-3′), a 0.2 µM TaqMan probe (5′ FAM - CGCCTGCACTGTCGTCATTAGTTA - TAMRA 3′), 0.2 mM each dNTP, 1 units of DNA polymerase, 1× PCR buffer, and 2 mM MgCl2. qPCR was carried out using a cycling sequence of 95°C for 2 min followed by 35 cycles of 95°C for 30 sec, 55°C for 30 sec and 68°C for 1 min, which was then followed by a final extension of 68°C for 7 min. To normalize the qPCR result, amplification of a housekeeping gene β-actin was performed for each sample with a previously reported primer set and dual-labeled probe [32]. After confirmation of the approximately equal amplification efficiencies of the RT-qPCR assay for both IAPV and β-actin (Figure S2), the concentration of IAPV in different tissues was interpreted using the comparative Ct method (ΔΔCt Method). The mean value and standard deviations of triplicate measurements of IAPV in each tissue was normalized using the Ct value corresponding to the triplicate measurements of endogenous control, β-actin following the formula: ΔCt = (Average Ct _DWV_)−(Average Ct _β-actin_). The hemolymph, with the lowest virus level of IAPV, was chosen as a calibrator. Each of the normalized target values was subtracted by the normalized value of the calibrator to yield ΔΔCt. The concentration of IAPV in each tissue was calculated using the formula 2^−ΔΔCt^ and expressed as the fold-change.

### In situ hybridization

Purified IAPV amplicons from primer pair IAPVF1/R1were incorporated into a pCR2.1 TA cloning vector (Invitrogen) which has a T7 site downstream of the insert and the orientation of the inserts was determined by sequence analysis. Probe complementary of genomic RNA of IAPV was generated from linearized plasmid using DIG-RNA Labeling Kit (T7) (Roche Applied Science, Indianapolis, IN). Eggs and queen tissues, including spermathecae, ovaries and gut, were subjected to dehydration by successive incubation in ethanol (70%, 95% and 100%) and xylol (2×5 min each) and then embedded in paraffin. Paraffin sections were cut ∼3–5 micron thick and mounted on poly-L-lysinated slides and stored at 4°C overnight. The sections were then rehydrated through a descending concentration of ethanol (100%, 95% and 70%), dewaxed in xylol, treated with proteinase K (10 ug/ml) for 30 minutes, and acetylated with 0.33% (v/v) acetic anhydride in 0.1 M triethanolamine-HCl (pH 8.0) for ten minutes prior to hybridization.

The sections were prehybridized in prehybridization solution (50% formamide, 5× SSC, 40 ug/ml salmon sperm) at 58°C for 2 hours and incubated in hybridization buffer with Dig-labeled IAPV probe solution to a concentration of 100–200 ng/ml probe in pre-hybridization solution at 58°C overnight. Negative control reactions included regular dUTP instead of DIG-labeled viral probe. After hybridization, the sections were washed in low stringency wash solution (2× SSC, 0.1% SDS) at room temperature for 5 minutes and washed twice in high stringency wash solution (0.1× SSC, 0.1% SDS) at 52°C for 15 minutes, and finally incubated with alkaline phosphatase (AP)-labeled sheep anti-DIG antibody conjugate. The hybridization signals were detected with alkaline phosphatase (AP)-labeled sheep anti-DIG antibody conjugate (Roche Applied Science). The conjugate solution was added to the dry sections and incubated at 4°C for 2 hours in a humid chamber. Color development was performed by adding the buffer solution containing nitroblue tetrazolium (NBT) and 5-bromo-4-chloro-3-indoyl phosphate (BCIP) to the tissue sections and incubating for 3–6 hours at room temperature with protection from light. Dark purple to blue coloring suggested the presence of the virus where the DIG-labeled probe bound directly to the viral RNA, while pink staining was shown in negative controls where no IAPV probe was included.

### Whole viral genome sequencing and phylogeny

To determine the levels of genetic diversity of IAPV, the complete genome sequences of IAPV strains from infected bees collected in MD, CA, and PA were determined by combining primer walking and long-range RT-PCR amplification using Invitrogen SuperScript One-Step RT-PCR System for Long Templates. The seven overlapping fragments of IAPV were amplified simultaneously. The sequences of the genome termini were determined by Invitrogen 3′ and 5′ RACE systems. The primers used to amplify overlapping long RT-PCR fragments and 3′ and 5′-RACE nested PCR were shown in Figure S1. The information regarding sequences and genomic positions of primers used in this study is shown in Table 1. Overlapping sequences were assembled into complete virus genomes using SeqMan (DNASTAR, Madison, WI, USA).

**Table 1:** IAPV primers used in the study.

IAPV (NC_009025)	Primers	Product Size (bp)	Genome Position	Reference
IAPV-F1 IAPV-R1	5′- gcggagaatataaggctcag-3′ 5′- cttgcaagataagaaaggggg-3′	586	23–608	Di Prisco et al. (2009)
IAPV-F2 IAPV-R2	5′- gctcagctaggatgacacg -3′ 5′- catgatgccctttgcagag -3′	1781	37–1817	This study
IAPV-F3 IAPV-R3	5′- ggatatgccagaagttgatcc -3′ 5′- caaagtaacttcatcagtag -3′	2185	1694–3878	This study
IAPV-F4 IAPV-R	5′- ctctgcaaagggcatcatg -3′ 5′- cattaatgatgagcggcgag -3′	2841	1798–4639	This study
IAPV-F5 IAPV-R5	5′- agctggagctacaactggc -3′ 5′- atggtaatgtccagcttcgt -3′	2299	4657–6949	This study
IAPV-F6 IAPV-R6	5′- taccatgcctggcgattcac -3′ 5′- gcaggacattaatgtactatatccag -3′	2821	6608–9428	This study
5′-RACE-R1	5′-cttgcaagataagaaaggggg-3′		589–609	This study
5′-RACE-R2	5′- tcaacaggtcccgggttttc-3′		1062–1082	This study
3′-RACE-F1	5′- ctacaaggcgaatcacgct -3′		9276–9296	This study
3′-RACE-F2	5′- gcaggacattaatgtactatatccag -3′		9403–9428	This study

The entire genome sequences of IAPV isolates from this study, as well as IAPV strains identified in Australia, China, and Israeli and previously deposited in GenBank were compared with the first reported strain of IAPV (GenBank Accession# NC_009025) in order to get a clear global picture of genetic diversity of IAPV strains. A phylogenetic tree was generated using all available complete genome sequences of IAPV. The sequence of *KBV* (GenBank Accession# NC_004807) was used as an outgroup to root the tree. Phylogenetic analysis was conducted in MEGA4 [36]. A tree was built using the Neighbor-Joining method and the reliability of the phylogenies was assessed by bootstrap replication (N = 1000 replicates). Node labels correspond to bootstrap support and those values >50% were regarded as providing evidence of phylogenetic grouping.

### Microarray hybridization and qRT-PCR validation

The global host responses of honey bees to IAPV infection in both adult and brood stages were investigated using microarray analysis. Adult worker bees (nurse bees inside the hive) and brood (4^th^ and 5^th^ instar larvae prior to capping) were collected from three colonies that were confirmed by RT-PCR to be infected with IAPV. The ubiquitous presence of DWV in both IAPV-positive and IAPV-negative bees was considered to be a background infection. Total RNAs from 10 IAPV-infected and 10 uninfected workers as well as 10 IAPV-infected and 10 uninfected brood were individually reverse transcribed into cDNA using Superscript III reverse transcriptase (Invitrogen) with random hexamers. The cDNA was labeled with Cytidine 3 (Cy3) and Cytidine 5 (Cy5), respectively, and reversed for the dye-swap analysis. The Cy5- or Cy3-labeled cRNA were mixed in the same amount and hybridized to honey bee oligonucleotide microarray slides fabricated at the University of Illinois. Slides were hybridized, washed, dried, and scanned using methods previously described [37]. The signal intensities were normalized based on the mean signal intensity across all genes on the arrays. The signal-to-noise ratio (SNR = <signal mean – background mean</<background standard deviation>] was then calculated for each spot to discriminate true signals from noise. Only spots with an SNR equal to or greater than 2.0 were considered positive. All negative, poor and empty spots were flagged and discarded. The normalized data were analyzed using Partek Genomics Suite Version 6.4 (Partek Inc., St. Louis, MO). Principal component analysis (PCA) and hierarchical clustering analysis were conducted with Partek with default settings. The fold changes of each gene expression in response to IAPV infection were calculated against the uninfected samples (negative control). Statistically significant genes were identified using mixed model analysis of variance (one-way ANOVA) with the Benjamini & Hochberg false discovery rate set to ≤0.05. The genes that displayed fold-changes of more than 1.5 (False Discovery Rate adjusted ρ value≤0.05) and had putative *D. melanogaster* orthologs were analyzed by

DAVID Bioinformatics Resources 6.7 (http://david.abcc.ncifcrf.gov), and GO browser and search engine AmiGO (http://www.geneontology.org) to define identify enriched biological themes in gene lists of both adult and brood. Additionally, the genes homologous to the their *Drosophila* gene counterparts were further analyzed for canonical pathways, biological functions/diseases, and functional molecular networks by Ingenuity Pathway Analysis (IGA) (Ingenuity Systems, Redwood City, CA). The Fisher's exact test was used to calculate a ρ- value to determine the probability that the association between the gene in the dataset and the predefined pathways and functional categories in the Ingenuity Pathway Knowledge Base is due to random chance alone.

A list of 20 genes involved in host immune responses were validated by SYBR Green real-time qRT-PCR in IAPV infected adult bees. The primers used in qRT-PCR are included in Table 2. The ΔΔCt method was chosen for interpretation of gene expression in response to IAPV infection following the same procedures described above. The approximately equal amplification efficiencies of the RT-qPCR assay for housekeeping gene β-actin and target immune genes were confirmed individually (the slope of normalized Ct vs. log input RNA≤0.1). The data output of each gene was expressed as a fold-change indicating whether the expression of the target gene in IAPV infected bees was up-regulated or down-regulated compared to the expression of the same gene in uninfected bees.

**Table 2:** Primers of immune genes in the study.

Immune Gene	GeneBank Accession #	Primers	Product Size (bp)	Reference
Cbl	XM_395448.3	F: 5′-gaggtaaggacgatcccaca-3′ R: 5′-ttcgtagcaaattcgtgcag-3′	184	This work
Stam	XM_623536.2	F: 5′-ggataggaggcatgcacagt-3′ R: 5′-agatggaccacctccaacag-3′	238	This work
Stat	XM_397181.3	F: 5′-attttgcaacacagccacaa-3′ R: 5′-ggtgcaccatttcctcctaa-3′	241	This work
Pias	XM_623536.2	F: 5′-gcgagttgcgatacaaacaa-3′ R: 5′-ccagcaaaaccaagaagcat-3′	190	This work
Hopscotch	XM_001121783	F: 5′-ttgtgctcctgaaaatgctg-3′ R: 5′-aacctccaaatcgctctgtg-3′	180	This work
GβL	XM_393223.3	F: 5′-cgagcctacgcgtcttaatc-3′ R: 5′-gacccatcgttttgcttcat-3′	184	This work
Mo25	XM_393376.3	F: 5′-tgcctctgttcggaaagtct-3′ R: 5′-tgggcaacaacaatatctgc-3′	202	This work
eIF4B	XM_624287.2	F: 5′-tcaaacaaggaatccgacct -3′ R: 5′-catttacaacagccccacaa -3′	186	This work
Dmel	XM_392604.3	F: 5′-ttttgggctgttttcaacaa-3′ R: 5′-agctgcaagcaccatttctt-3′	186	This work
Raptor	XM_624057.2	F: 5′-cggaagaggatgattggaaa-3′ R: 5′-tggatcaacgccaacattta-3′	238	This work
Ras	XM_395469.3	F: 5′-gcgtgtgagtgtcaagctgt-3′ R: 5′-ccttcaaatccagctcttgc-3′	248	This work
Phl	XM_396892.2	F: 5′-cctgctttatgccaccaagt-3′ R: 5′-gaacgtggatgcctttgatt-3′	167	This work
TII	XM_624039.1	F: 5′-ctggaattccgcacgtttat-3′ R: 5′-cagcttcctccgaacttgtc-3′	195	This work
Pointed	XM_396368.3	F: 5′-gaaccgttctacgccgatta-3′ R: 5′-ctgattctcgtcttggcaca-3′	240	This work
Corkscrew	XM_003249806	F: 5′-ttgctgcttctcttgcttca-3′ R: 5′-gttctgcttgcattcgttga-3	285	This work
Past1	XM_396463.3	F: 5′-ttttgatgcaaaacccatg-3′ R: 5′-tggacgaaactgcttgtttg-3′	211	This work
CG1115	XM_001120612	F: 5′-acagcagcagctgaaattga-3′ R: 5′-ttggataaggaattgcaggaa-3	241	This work
CG6259	XM_392468.3	F: 5′-gccgacgaagtacaagaagc-3′ R: 5′-tttgtggcaaaccaaattca-3′	229	This work
Rabenosyn	XM_392585.3	F: 5′-cgggaatcggtcttacaaaa-3′ R: 5′-tgttgcgaagcttcttccat-3′	229	This work
EGFR	XM_003249561	F: 5′-gtgaacagtgcgaagacgaa-3′ R: 5′-ggaacaatacggttcgctgt-3′	248	This work

### Effect of silencing putative viral suppressor of RNAi

Complete predicted protein sequences of IAPV (NC_009025.1), along with other honey bee viruses, including KBV (NC_004807.1), ABPV (NC_002548.1), CrPV (NC_003924), and DCV (NC_001834) were retrieved from GenBank and scanned for the DvExNPGP sequence motif where the upstream sequences of the DvExNPGP motif was reported to encode a RNAi suppressor [13]. A DvExNPGP sequence motif was identified in IAPV and the upstream sequences of the DvExNPGP motif at the 5′ terminus of the IAPV genome was therefore assumed to be a putative IAPV-encoded suppressor (Figure S1). siRNA corresponding to upstream sequences of DvExNPGP was designed using online siRNA design tool siDirect version 2.0 (http://sidirect2.rnai.jp/). The sequences of the siRNAs used in this study are as follows: 5′-UACAACUUAUUCAAGAAUCCA-3′ and 5′- GAUUCUUGAAUAAGUUGUACC-3′. The chemically modified, 21-mer, double-stranded and in vivo ready siRNAs were synthesized in a 250 nmol scale by Ambion Life Technologies (CA, USA). The impact of siRNA corresponding to a putative IAPV-encoded VSR on IAPV replication was investigated by a laboratory cage study as described previously [38]. Briefly, the frames with emerging brood were removed from the colonies left untreated for *V. destructor* for 2–3 moths and identified with IAPV infection by RT-PCR assay, and newly emerged bees were collected the following day. Forty bees were placed in each rearing cage for the assay. A scintillation vial filled with a 1∶1 ratio sucrose-water solution was inverted over the top of the rearing cup as provision for the caged bees. The caged bees were divided into four groups: Group-I consisting of siRNA-treated IAPV-infected bees not exposed to parasitic mites *V. destructor*, Group-II consisting of untreated IAPV-infected bees not exposed to *V. destructor*, Group-III consisting of treated IAPV-infected bees challenged by *V. destructor*, and Group-IV consisting of untreated IAPV-infected bees challenged by *V. destructor*. The *Varroa* mites used in the study were collected from a colony that was heavily infested with mites; both honey bees and mites were shown to be infected with IAPV using RT-PCR assay. Twelve *Varroa* mites were introduced to each cage to create 30% Varroa mite infection. For groups receiving siRNA feeding, siRNA was mixed with sugar water in the scintillation vials, resulting in a 50 nM/ul working concentration of siRNA. Ten experimental bees along with 3 mites were collected at day 1, day 3, day 5, and day 7 post-treatment. The assay was repeated three times. The effect of silencing putative VSR on IAPV replication was analyzed by quantifying the titer of negative-stranded RNA of IAPV in bees from each group by real time RT-qPCR following the method described above.

### Ethics statement

No specific permits were required for the described studies. Observations were made in the USDA-ARS Bee Research Laboratory apiaries, Beltsville, Maryland, USA; therefore, no specific permissions were required to be obtained for these locations. The apiaries are the property of the USDA-ARS and are not privately-owned or protected in any way. Studies involved the European honey bee (*Apis mellifera*), which is neither an endangered nor protected species.

## Supporting Information

Figure S1 **IAPG Genome Organization and overlapping PCR fragments spanning the entire viral genome.** (A) Like other members of the dicistroviruses, the genome of IAPV is monopartite and bicistronic with replicase proteins (Hel, Pro, RdRp) encoded by a 5′-proximal Open Reading Frame (ORF) and capsid proteins (VP1-4) by a 3′-proximal ORF. The position of the sequence motif, DvExNPGP, is shown. (B) Schematic diagram indicates the relative locations of overlapping PCR fragments and cDNA ends. The full-length IAPV genomes were sequenced using a combination of long-template RT-PCR amplification and methods for rapid amplification of 5′ and 3′ cDNA ends (5′RACE and 3′ RACE).
(TIF)

Figure S2 **Amplification efficiencies of IAPV and β-actin.** The difference between the Ct value of IAPV and Ct value of β-actin (ΔCt) was plotted versus the log of six 10-fold dilutions of total RNA. The plot of log total RNA input versus ΔCt has a slope less than 0.1, indicating that the efficiencies of the two amplicons were approximately equal. Therefore, the ΔΔCt calculation for the relative quantitation of IAPV in this study was valid.
(TIF)

Figure S3 **Microarray data validation.** A) Principal component analysis (PCA) scatter plot. PCA analysis of all differentially regulated genes clearly separates the two different data sets IAPV positive (IAPV^+^) and IAPA negative (IAPV^−^) for both adults and brood (4^th^ and 5^th^ instar larvae, prepupae and white-eyed pupae). B) Unsupervised hierarchical clustering of gene expression data. Hierarchical cluster analysis shows the differential expression of genes in both adults and brood in response to IAPV infection. C) Variance ratios from ANOVA (error set to 1). For both adults and brood, variance of treatment (IAPV infected VS. uninfected) was significantly higher than error (ρ<0.01). D) Volcano Plot. The volcano plots show a large group of up and down regulated genes in response to IAPV infection in adults and brood. Each dot represents one gene with detectable expression. The horizontal line marks the threshold (*p*≤0.05, adjusted using the Benjamini & Hochberg false discovery rate) for defining genes with altered expression. The vertical lines represent change ≥1.5 fold in expression and define genes as up-regulated (right) or down-regulated (left).
(TIF)

Table S1 **Functional annotation clustering of activated genes in response to IAPV infection in adults.**
(XLSX)

Table S2 **Functional annotation clustering of activated genes in response to IAPV infection in brood.**
(XLSX)
