# Supplementary figures and images for: Israeli Acute Paralysis Virus: Epidemiology, Pathogenesis and Implications for Honey Bee Health
Source: PLoS Pathog. 2014 Jul 31;10(7):e1004261. doi: 10.1371/journal.ppat.1004261 (PMC4117608; doi:10.1371/journal.ppat.1004261)

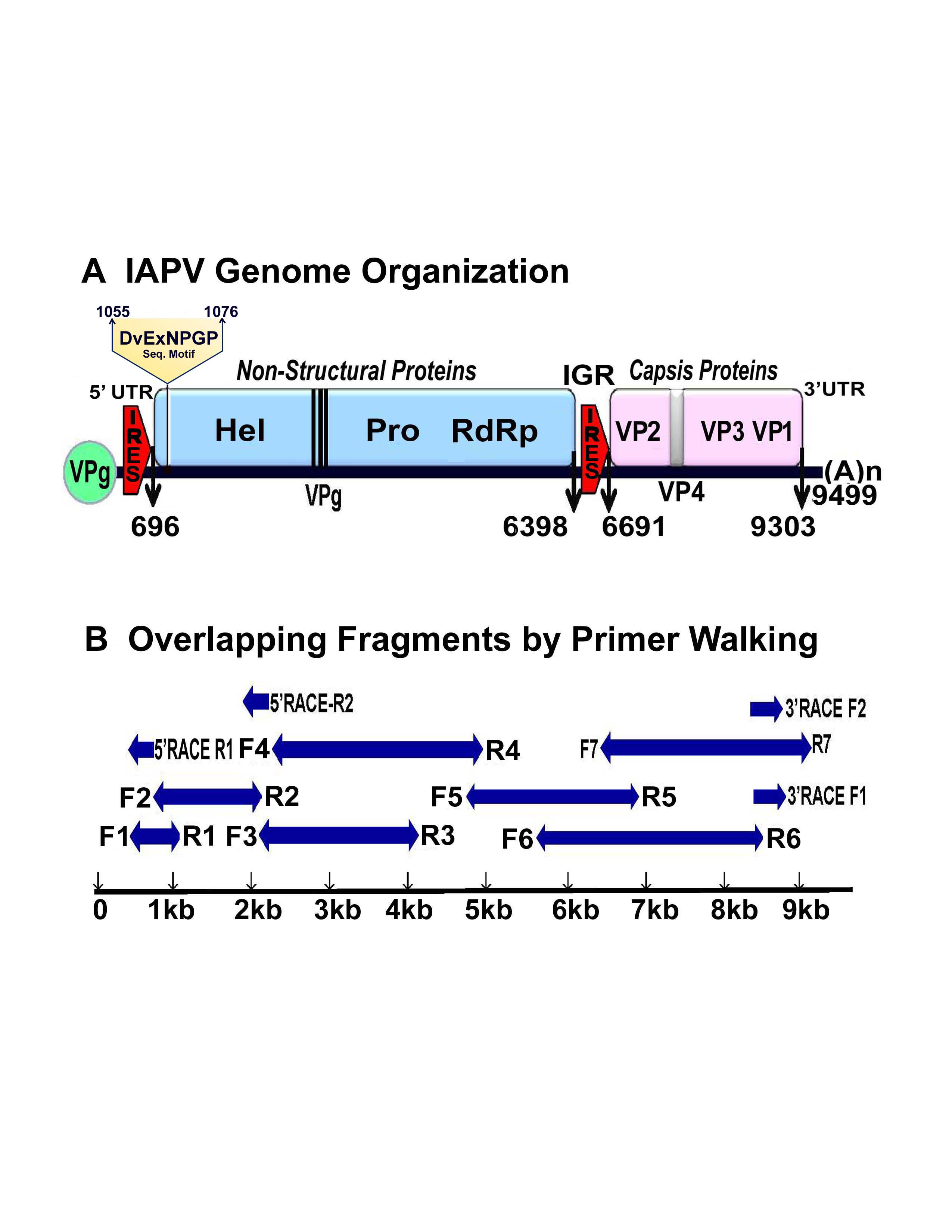

Supplement: Figure S1 — IAPG Genome Organization and overlapping PCR fragments spanning the entire viral genome. (A) Like other members of the dicistroviruses, the genome of IAPV is monopartite and bicistronic with replicase proteins (Hel, Pro, RdRp) encoded by a 5′-proximal Open Reading Frame (ORF) and capsid proteins (VP1-4) by a 3′-proximal ORF. The position of the sequence motif, DvExNPGP, is shown. (B) Schematic diagram indicates the relative locations of overlapping PCR fragments and cDNA ends. The full-length IAPV genomes were sequenced using a combination of long-template RT-PCR amplification and methods for rapid amplification of 5′ and 3′ cDNA ends (5′RACE and 3′ RACE). (TIF) [file ppat.1004261.s001.tif]

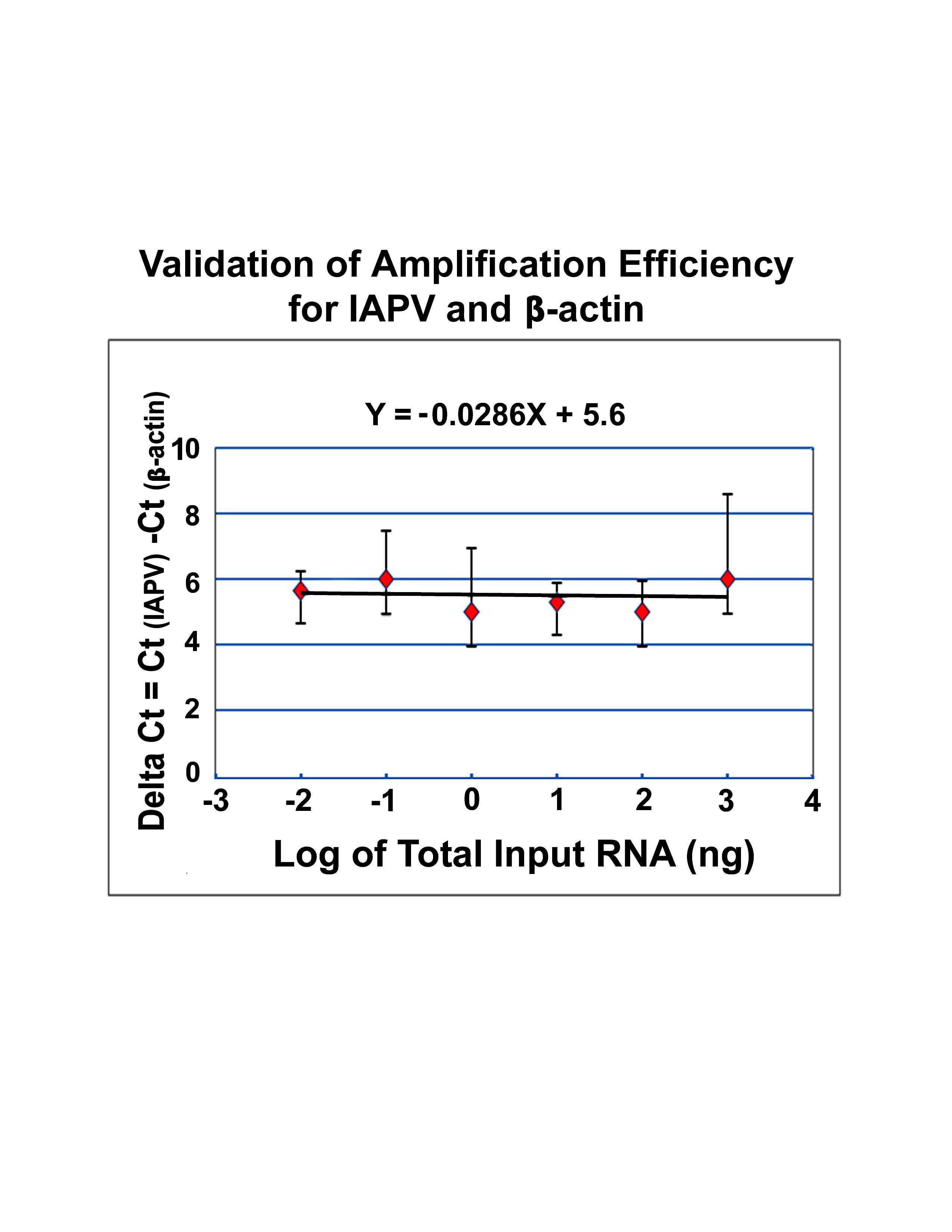

Supplement: Figure S2 — Amplification efficiencies of IAPV and β-actin. The difference between the Ct value of IAPV and Ct value of β-actin (ΔCt) was plotted versus the log of six 10-fold dilutions of total RNA. The plot of log total RNA input versus ΔCt has a slope less than 0.1, indicating that the efficiencies of the two amplicons were approximately equal. Therefore, the ΔΔCt calculation for the relative quantitation of IAPV in this study was valid. (TIF) [file ppat.1004261.s002.tif]

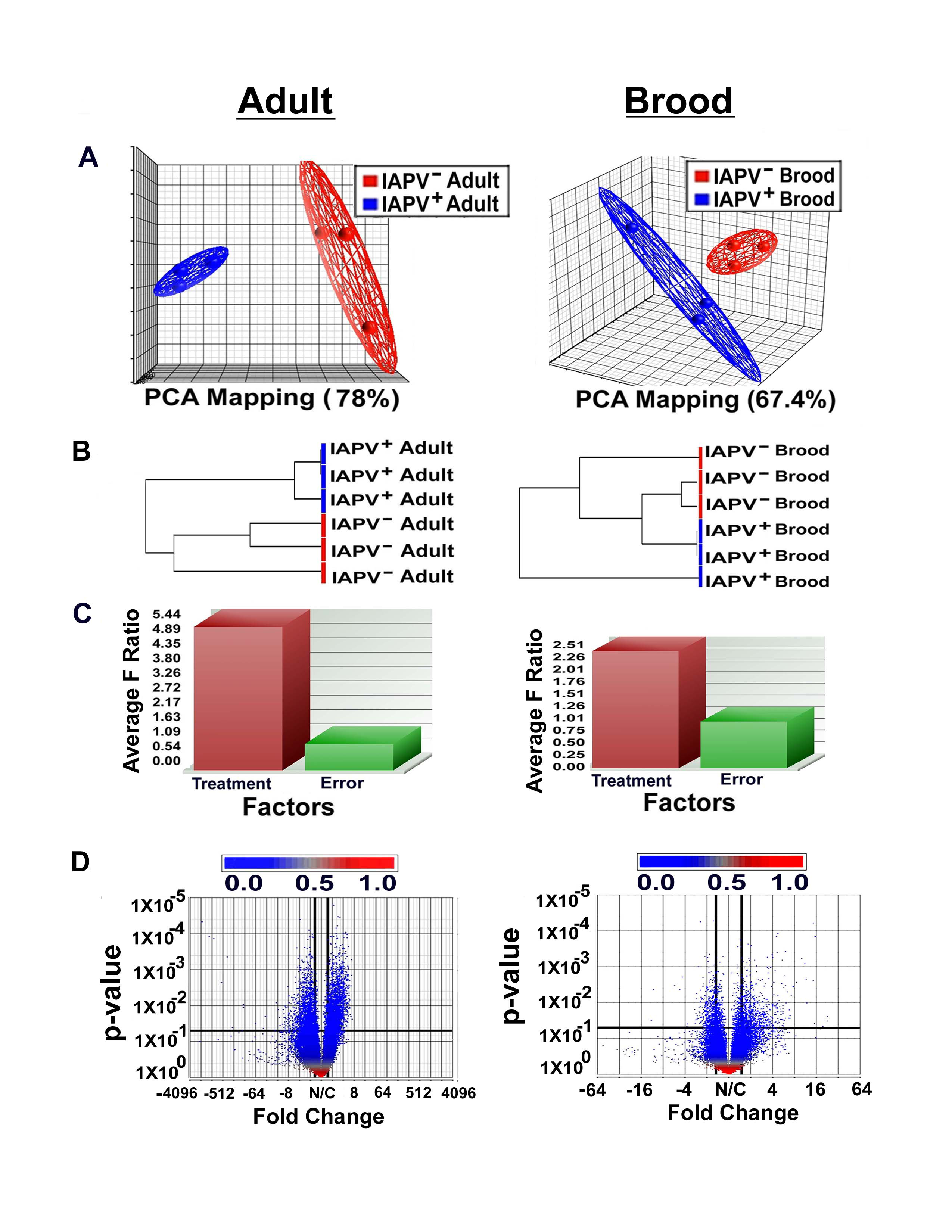

Supplement: Figure S3 — Microarray data validation. A) Principal component analysis (PCA) scatter plot. PCA analysis of all differentially regulated genes clearly separates the two different data sets IAPV positive (IAPV+) and IAPA negative (IAPV−) for both adults and brood (4th and 5th instar larvae, prepupae and white-eyed pupae). B) Unsupervised hierarchical clustering of gene expression data. Hierarchical cluster analysis shows the differential expression of genes in both adults and brood in response to IAPV infection. C) Variance ratios from ANOVA (error set to 1). For both adults and brood, variance of treatment (IAPV infected VS. uninfected) was significantly higher than error (ρ<0.01). D) Volcano Plot. The volcano plots show a large group of up and down regulated genes in response to IAPV infection in adults and brood. Each dot represents one gene with detectable expression. The horizontal line marks the threshold (p≤0.05, adjusted using the Benjamini & Hochberg false discovery rate) for defining genes with altered expression. The vertical lines represent change ≥1.5 fold in expression and define genes as up-regulated (right) or down-regulated (left). (TIF) [file ppat.1004261.s003.tif]
